# Intensive care unit caseload and workload and their association with outcomes in critically unwell patients: a large registry-based cohort analysis

**DOI:** 10.1186/s13054-024-05090-z

**Published:** 2024-09-14

**Authors:** Paul Zajic, Teresa Engelbrecht, Alexandra Graf, Barbara Metnitz, Rui Moreno, Martin Posch, Andrew Rhodes, Philipp Metnitz

**Affiliations:** 1 Department of Anaesthesiology and Intensive Care Medicine, Medical University of Graz, Auenbruggerplatz 5, 8036 Graz, Austria; 2 Center for Medical Data Science, Medical University of Vienna, Vienna, Austria; 3 Austrian Center for Documentation and Quality Assurance in Intensive Care, Vienna, Austria; 4 Hospital de São José, Unidade Local de Saúde São José, Lisbon, Portugal; 5 Centro Clínico Académico de Lisboa, Lisbon, Portugal; 6 Faculdade de Ciências da Saúde, Universidade da Beira Interior, Lisbon, Portugal; 7 Adult Critical Care, St. George’s University Hospitals NHS Foundation Trust, St. George’s University of London, London, UK

**Keywords:** Critical care, Inpatients, Workload, Facilities and services utilization, Mortality

## Abstract

**Background:**

Too high or too low patient volumes and work amounts may overwhelm health care professionals and obstruct processes or lead to inadequate personnel routine and process flow. We sought to evaluate, whether an association between current caseload, current workload, and outcomes exists in intensive care units (ICU).

**Methods:**

Retrospective cohort analysis of data from an Austrian ICU registry. Data on patients aged ≥ 18 years admitted to 144 Austrian ICUs between 2013 and 2022 were included. A Cox proportional hazards model with ICU mortality as the outcome of interest adjusted with patients’ respective SAPS 3, current ICU caseload (measured by ICU occupancy rates), and current ICU workload (measured by median TISS-28 per ICU) as time-dependent covariables was constructed. Subgroup analyses were performed for types of ICUs, hospital care level, and pre-COVID or intra-COVID period.

**Results:**

415 584 patient admissions to 144 ICUs were analysed. Compared to ICU caseloads of 76 to 100%, there was no significant relationship between overuse of ICU capacity and risk of death [HR (95% CI) 1.06 (0.99–1.15), *p* = 0.110 for > 100%], but for lower utilisation [1.09 (1.02–1.16), *p* = 0.008 for ≤ 50% and 1.10 (1.05–1.15), *p* < 0.0001 for 51–75%]. Exceptions were significant associations for caseloads > 100% between 2020 and 2022 [1.18 (1.06–1.30), *p* = 0.001], i.e., the intra-COVID period. Compared to the reference category of median TISS-28 21–30, lower [0.88 (0.78–0.99), *p* = 0.049 for ≤ 20], but not higher workloads were significantly associated with risk of death. High workload may be associated with higher mortality in local hospitals [1.09 (1.01–1.19), *p* = 0.035 for 31–40, 1.28 (1.02–1.60), *p* = 0.033 for > 40].

**Conclusions:**

In a system with comparably high intensive care resources and mandatory staffing levels, patients’ survival chances are generally not affected by high intensive care unit caseload and workload. However, extraordinary circumstances, such as the COVID-19 pandemic, may lead to higher risk of death, if planned capacities are exceeded. High workload in ICUs in smaller hospitals with lower staffing levels may be associated with increased risk of death.

**Supplementary Information:**

The online version contains supplementary material available at 10.1186/s13054-024-05090-z.

## Background

Care for critically unwell patients is a complex interplay of processes requiring significant material, economic, and human resources [1–4] over periods of time. If any of these resources is strained, pressure on those remaining is built up and decisions must be made on whom to supply them to [5]. The COVID-19 pandemic has led to public recognition that all parts of this construct can relatively rapidly be overwhelmed when demand or necessity exceed capacity [6, 7]. It stands to reason that high demand and workload during normal working conditions may influence outcomes as well [8].

In general, high workload may lead to inter-personal conflict [9], medical error [10], and limited quality and safety of care [11]. Conversely, higher exposure to critically unwell patients may lead to higher experience in their treatment and may foster well-designed processes. It has been demonstrated that higher compared to lower overall patient volumes are associated with better outcomes in several critically ill patient groups [12] and mechanically ventilated patients [13].

Based on harms and benefits conveyed by work intensity and patient volumes, we hypothesise that a relationship between ongoing caseload, ongoing workload, and outcomes exists that describes optimal intensive care utilisation. Conversely, too high or too low patient volumes and work amounts may overwhelm health care professionals and obstruct processes or lead to inadequate personnel routine and process flow. Such knowledge could aid decision-making, capacity building, and patient disposition both during routine work and in surge situations.

Studies on association of intensive care unit census, i.e., bed occupancy rates, at patients’ admission and outcomes conducted before the COVID-19 pandemic have mostly demonstrated no [14] or relatively modest associations [15–17], while admission to strained units for COVID-19 has been found associated with increased risk of death [6, 18, 19]. However, care for critically unwell patients is a process usually spanning over at least several days [20]. Little is known about how work intensity over time may affect outcomes.

In this study we aim to evaluate whether an association between ongoing caseload, ongoing workload, and outcomes exists in intensive care medicine. To do so, we seek to identify possible associations between risk of mortality adjusted for baseline severity of illness in critically unwell patients treated in intensive care units, current occupancy rates of the respective intensive care units, and current staff workload.

## Materials and methods

### Study design and data source

This study was conducted as a retrospective analysis of registry data collected in the Austrian Center for Documentation and Quality Assurance in Intensive Care Medicine (ASDI) database. Participating ICUs contributed data based on yearly contractual agreements in accordance with national legislation that requires structured reporting of key data. An in-depth description of database contents and detailed variable definitions were published previously [21, 22].

The dataset encompassed ICU-related data, i.e., type of ICU, planned number of beds, hospital care level (see below), and patient-related data, i.e., sociodemographic data (age, sex, chronic conditions, etc.), reasons for ICU admission (recorded according to a predefined list of diagnoses), severity of illness (measured by Simplified Acute Physiology Score 3 (SAPS 3) [20, 23]), level of provided care (measured by Simplified Therapeutic Intervention Scoring System (TISS-28) [24]), length of ICU and hospital stay, and outcome data (survival status at ICU and hospital discharge).

### Study setting

Health care provision in Austria, including intensive care units (ICUs), is regulated according to a national structure plan for health (Österreichischer Strukturplan Gesundheit). Intensive care for adults may be provided at local (primary), regional (secondary), special-purpose (specialised), and central (tertiary) hospitals.

ICUs are accordingly categorised in levels 1, 2 and 3, similar to international understanding of ICU grading [4]. Austria has previously been found to have above-average intensive care bed capacity in Europe with approximately 21.8 critical care beds per 100,000 inhabitants [25]. Intermediate care units (IMCU) exist in some institutions; the national structure plan for health suggests these to be connected to ICUs.

Any ICU must be led by a specialist physician qualified in intensive care medicine (stem-specialties Anaesthesiology, Internal Medicine, and others). A specialist physician needs to be present within the hospital continuously for all ICUs and must be present within the unit for level 3 ICUs. Nursing care is provided by diploma-grade nurses, of whom at least half must have undergone intensive care specialisation training. Mandatory total nursing staff levels per unit are defined by nurse-to-bed ratios of ≥ 2:1, ≥ 2.5:1, and ≥ 3:1 for ICUs of level 1, 2, and 3, respectively. Allied health care professional staffing is not strictly regulated.

### Patient selection

Adult patients (age 18 years and above) admitted to participating Austrian ICUs between January 1st, 2013, and December 31st, 2022, were included in this study. Patients, in whom SAPS 3 or outcome data were missing, were excluded from analyses.

### Outcomes

The primary outcome of interest for this study was mortality in the ICU. This outcome was chosen since the factors in question only pertain to patients’ respective stays in intensive care.

### Variables

Patients’ individual severity of illness was modelled using numeric SAPS 3 score documented upon ICU admission. Current ICU caseload and ICU workload were modelled in 8-h time blocks (8:00–15:59, 16:00–23:59, 00:00–07:59) to represent shift patterns, allow for acceptable granularity and model performance, and reflect differences in caseload and workload over the day [26, 27].

Current ICU caseload in each time block, represented by bed occupancy rate, was defined as the highest number of patients admitted to an ICU documented within this time block divided by the total number of planned beds reported for this ICU in the respective year. Current ICU workload in each time block was defined as the median TISS-28 score [24] of patients present at an ICU other than the respective patient within this time block. This approach was chosen to avoid confounding with individual patients’ therapeutic intervention level.

### Statistical analysis

Descriptive data were presented as median and interquartile ranges (IQR) or absolute number (n) and percentage (%), unless specified otherwise.

For primary analysis aiming at the instantaneous risk of death associated with current ICU caseload and current ICU workload, a Cox proportional hazards model with ICU mortality as the outcome of interest was constructed. The model was adjusted with current ICU caseload (as described above), and current ICU workload (as described above), type of day (working day or non-working day), and time of day (8:00–15:59; 16:00–23:59; 00:00–7:59) as time-dependent covariables. Further covariables used were patients’ respective SAPS 3, patient sex, year of ICU discharge, hospital care level, and type of ICU. ICU identifiers were used as a clustering variable.

To assess the stability of our model, sensitivity analyses were performed. To investigate a potentially more complex interplay between workload and caseload, the primary model was reconstructed including an interaction term between current ICU workload (as described above) and ICU current caseload (as described above). To address potential delayed effects of caseload and workload, the primary model was reconstructed with ICU caseload (as described above) and ICU workload (as described above) modelled with their respective median values over up to the last three days (i.e., nine time blocks) of a patient’s ICU stay as time-dependent covariables (“moving medians”). The timespan of three days was chosen to encompass common scheduling and rostering cycles (especially weekends). Furthermore, models were repeated using ICU identifiers as covariables rather than clustering variables. In these models, hospital care level and type of ICU could not be included as covariables.

To explore potential differences in results within certain structures or timeframes, models were re-calculated in subgroups stratified by type of ICU (i.e., medical, surgical), hospital care level (i.e., primary, secondary, specialised, tertiary), and pre-COVID or intra-COVID period (i.e., 2013–2019, 2020–2022). To ascertain the robustness of subgroup findings, the primary model was repeated including interaction terms between current ICU caseload and current ICU workload with the aforementioned group variables.

Due to the retrospective and exploratory character of the study, a p-value smaller than 0.05 was considered statistically significant and no correction for multiplicity was applied. Furthermore, models were repeated using ICU identifiers as covariables rather than clustering variables. In these models, hospital care level and type of ICU could not be included as covariables.Analyses were conducted using R (4.3.1) [28] with packages dplyr (1.1.2), stats (4.3.1), tidyr (1.3.0), tibble (3.2.1), ggpubr (0.6.0), lubridate (1.9.2), flextable (0.9.2), ggplot2 (3.4.3), survival (3.5.7), survminer (0.4.9), forcats (1.0.0), purr (1.0.2), stringr (1.5.0), utils (4.3.1).

## Results

**Fig. 1:**
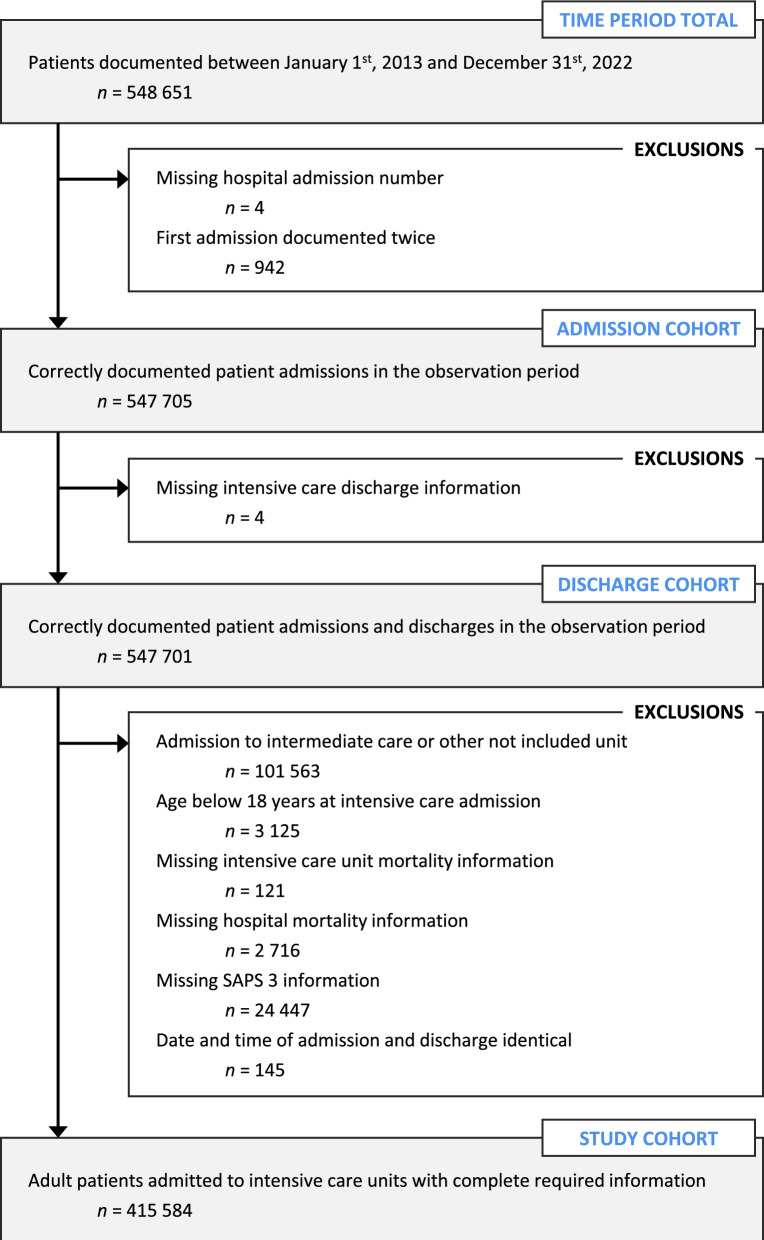
Study flow chart

Data on 415,584 patient admissions were retrieved and analysed in this study (Fig. 1). Median patient age was 69 (57–78) years, 242,870 (58.4%) patients were male, 42,243 (10.2%) died during ICU stay, and 62,982 (15.2%) died during hospital stay. See Table 1 for characteristics of the patient cohort.

**Table 1:** Patient characteristics upon admission to the intensive care unit, unadjusted outcomes, and measures of ICU caseload and ICU workload

	Overall cohort	Death in ICU	
		No	Yes
*n* of patients	415,584	373,341	42,243
Age (median, IQR)	69 (57–78)	68 (56–77)	73 (64–81)
Male sex (n, %)	242,870 (58.4%)	217,796 (58.3%)	25,074 (59.4%)
Type of admission (n, %)			
Medical	207,151 (49.8%)	176,066 (47.2%)	31,085 (73.6%)
Non-scheduled surgery	77,027 (18.5%)	68,626 (18.4%)	8401 (19.9%)
Scheduled surgery	129,571 (31.2%)	126,965 (34.0%)	2606 (6.2%)
ICU admission diagnosis (n, %)			
Respiratory disease	44,684 (10.8%)	37,297 (10.0%)	7387 (17.5%)
Cardiovascular disease	57,752 (13.9%)	49,675 (13.3%)	8077 (19.1%)
Neurologic disease	16,750 (4.0%)	14,542 (3.9%)	2208 (5.2%)
Other disease	49,672 (12.0%)	42,083 (11.3%)	7589 (18.0%)
Cardiovascular surgery	35,202 (8.5%)	33,907 (9.1%)	1295 (3.1%)
Abdominal surgery	41,259 (9.9%)	37,983 (10.2%)	3276 (7.8%)
Other surgery	56,621 (13.6%)	54,458 (14.6%)	2163 (5.1%)
Trauma	29,284 (7.0%)	27,310 (7.3%)	1974 (4.7%)
Other or unknown	84,360 (20.3%)	76,086 (20.4%)	8274 (19.6%)
SAPS 3 (median, IQR)	47 (38–59)	46 (37–56)	68 (58–79)
Length of stay (median, IQR)			
ICU	3 (2–6)	3 (2–6)	4 (2–10)
Hospital	13 (7–24)	14 (8–25)	7 (3–16)
Mortality (n, %)			
ICU	42,243 (10.2%)	0 (0.0%)	42,243 (100.0%)
Hospital	62,982 (15.2%)	20,739 (5.6%)	42,243 (100.0%)
Current ICU caseload [%] (median, IQR)	86 (71–100)	86 (71–100)	83 (71–100)
Current ICU workload (median, IQR)	31 (27–35)	31 (27–34)	33 (30–36)

Current ICU caseload was modelled as the median of ICU occupancy rates (defined as the highest number of patients admitted to an ICU in 8-h time blocks divided by the total number of beds reported for the respective ICU and year) over the course of patients’ ICU stays. Current ICU workload was modelled as the median of across-ICU therapeutic intervention scores (defined as median TISS-28 scores of all patients present at an ICU within 8-h time blocks) over the course of patients’ ICU stays

*ICU* intensive care unit, *IQR* inter-quartile range, *n* number, *SAPS* Simplified Acute Physiology Score, *TISS* Therapeutic Intervention Scoring System

In total, 144 ICUs (minimum 85, maximum 125 per year) contributed data used in this study. Of these ICUs, 50 (34.7%) were categorised as medical and 94 (65.3%) as surgical. Median bed capacity per ICU over the study time frame was 7 (6–8). See Table 2 for ICU characteristics.

**Table 2:** Intensive care unit characteristics and indicators of caseload and workload

	Intensive care units
*n*	144
ICU type (n, %)	
Medical	50 (34.7%)
Surgical	94 (65.3%)
Hospital level (n, %)	
Primary care hospital	48 (33.3%)
Secondary care hospital	52 (36.1%)
Specialised care hospital	17 (11.8%)
Tertiary referral hospital	27 (18.8%)
*n* of beds per ICU (median, IQR)	7 (6–8)
*n* of beds per ICU (n, %)	
< 8	75 (52.1%)
8–10	52 (36.1%)
11–13	14 (9.7%)
> 13	3 (2.1%)

*ICU* intensive care unit, *IQR* inter-quartile range, *n* number

**Fig. 2:**
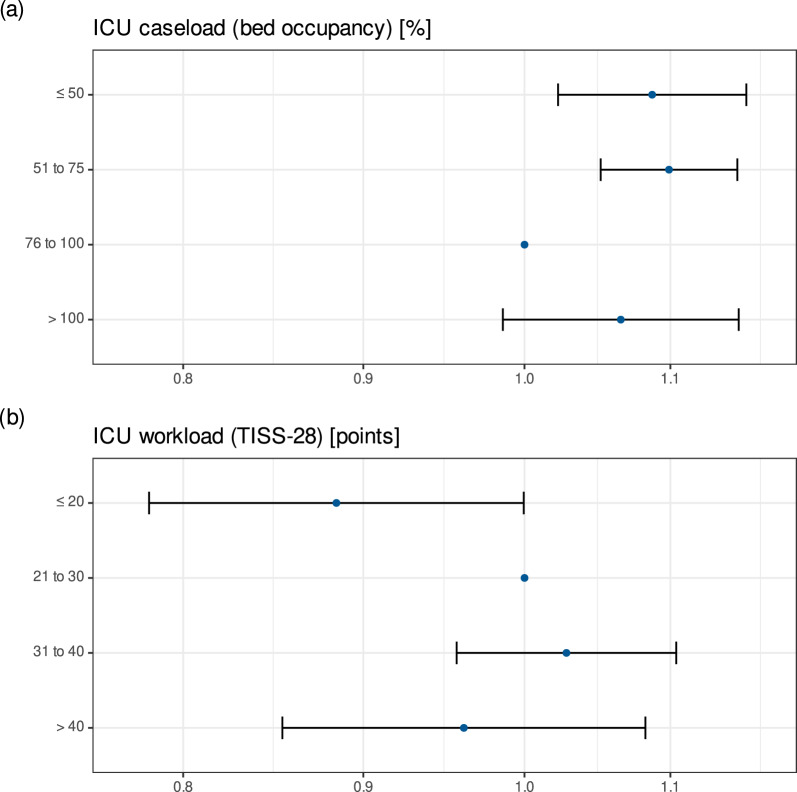
Results of Cox proportional hazards model with ICU mortality as the endpoint for (above) current ICU caseload represented by ICU bed occupancy rates and (below) current ICU workload represented by median TISS-28 scores per ICU. ICU = intensive care unit, TISS = Therapeutic Intervention Scoring System

The primary analysis model identified low current ICU caseload to be significantly associated with risk of death in the ICU (Table 3, Fig. 2a). Compared to the reference group of current ICU caseload from 76 to 100%, occupancy rates ≤ 75% were associated with significantly higher risk of ICU mortality [HR (95% CI) 1.09 (1.02–1.16), *p* = 0.008 for ≤ 50% and 1.10 (1.05–1.15), *p* < 0.0001 for 51–75%]. There was no significant relationship between overuse of ICU capacity and risk of death [HR (95% CI) 1.06 (0.99–1.15), *p* = 0.110 for > 100%].

**Table 3:** Cox proportional hazards model with ICU mortality as the endpoint. Current ICU caseload was defined as the highest number of patients admitted to an ICU in 8-h time blocks divided by the total number of beds reported for the respective ICU and year

	HR	95% CI		*p*
SAPS 3 [per point]	1.05	1.05	1.06	< 0.0001
Patient gender				
Female	1.00			
Male	0.91	0.89	0.93	< 0.0001
Hospital level				
Primary care hospital	1.00			
Secondary care hospital	1.03	0.91	1.16	0.675
Specialised care hospital	0.73	0.62	0.87	< 0.0001
Tertiary care hospital	0.91	0.74	1.11	0.351
ICU type				
Medical	1.00			
Surgical	0.73	0.65	0.83	< 0.0001
Year of discharge				
2013	1.00			
2014	0.92	0.86	0.99	0.028
2015	0.88	0.79	0.98	0.016
2016	0.98	0.86	1.11	0.696
2017	0.97	0.87	1.08	0.575
2018	1.00	0.89	1.13	0.938
2019	0.93	0.82	1.05	0.256
2020	1.12	0.99	1.26	0.077
2021	1.13	0.99	1.28	0.064
2022	1.03	0.92	1.15	0.575
Time of day				
08:00–15:59	1.00			
16:00–23:59	0.90	0.86	0.94	< 0.0001
00:00–07:59	0.49	0.47	0.52	< 0.0001
Calendar day				
Working day	1.00			
Non-working day	0.89	0.86	0.92	< 0.0001
Current ICU caseload (bed occupancy) [%]				
≤ 50	1.09	1.02	1.16	0.008
51–75	1.10	1.05	1.15	< 0.0001
76–100	1.00			
> 100	1.06	0.99	1.15	0.110
Current ICU workload (TISS-28) [points]				
≤ 20	≤ 20	0.88	0.78	0.99
21–30	1.00			
31–40	1.03	0.96	1.10	0.455
> 40	0.96	0.85	1.08	0.512

Current ICU workload was defined as the median of TISS-28 scores of all other patients present at an ICU within 8-h time blocks. Current ICU caseload and Current ICU workload were modelled as time-dependent covariables

*95% CI* 95% confidence interval, *HR* hazard ratio, *SAPS 3* Simplified Acute Physiology Score, *TISS* Therapeutic Intervention Scoring System

Low current ICU workload was also found to be associated with lower risk of death in the ICU (Table 3, Fig. 2b). Compared to the reference group of median TISS-28 scores within ICUs from 21 to 30, median values ≤ 20 were associated with significantly lower risk of ICU mortality [HR (95% CI) 0.88 (0.78–0.99), *p* = 0.049). Higher current ICU workloads were not found to be significantly associated with risk of death in the ICU. The model was evaluated to have satisfactory goodness-of-fit (c = 0.83).

Findings from the primary analysis model were generally stable across the above-described sensitivity analyses. The inclusion of an interaction term between current ICU workload and current ICU caseload did not yield significant results overall (Table S1 and Fig. S1 in the ESM). No significant longer-time effects of workload and caseload were seen in the model including “moving” median values over the last three days (Table S2 in the ESM). Using ICU identifiers as covariables instead of clustering variables did not notably alter results (Table S3 in the ESM).

Findings from subgroup analyses and interaction checking were generally similar to those from the primary model (Table S4–S14 in the ESM). Notable deviations were: current ICU caseload exceeding 100% of capacity was associated with significantly increased risk of death in the ICU between 2020 and 2022 [HR (95% CI) 1.18 (1.06–1.30), *p* = 0.001; HR (95% CI) 1.19 (1.06–1.34), *p* = 0.003 for interaction) and higher current ICU workload represented by median TISS-28 per ICU above 30 was associated with significantly higher risk of death in the ICU in primary care hospitals [HR (95% CI) 1.09 (1.01–1.19), *p* = 0.035 for 31–40, 1.28 (1.02–1.60), *p* = 0.033 for > 40 compared to 21–30].

## Discussion

In this large cohort study conducted in a comprehensive Austrian registry of ICU data, we demonstrate that in intensive care units with mandated staffing levels, high current caseload and workload are not generally associated with worsened outcomes. These assertions may not hold true for every type of intensive care unit and every circumstance. Here, we aim to highlight potential explanations for these findings that appear most relevant for planning and management of intensive care provision as potential leverage points for quality assurance.

Workload in ICUs is a “multidimensional and complex construct” and as such not necessarily straightforward in its depiction and investigation [29]. An obvious aspect of workload is variable bed occupancy in contrast to relatively fixed personnel numbers. Prior studies have investigated possible associations of ICU census at patients’ admission and outcomes.

A study conducted in 200 499 patients from 108 ICUs between 2002 and 2005 has not found such an association [14]. Another study in 264,401 patients admitted to 155 ICUs in the United States between 2001 to 2008 has found ICU census, both at admission and averaged over three days, to be associated with increased risk of mortality, especially when average measures of illness severity in the patient group is high [15]. In 149,310 patients admitted to 215 ICUs in the United Kingdom, a trend towards increased mortality with higher ICU strain has been found, again especially when patient acuity is high [17]. We have not found such a relationship in an analysis using an interaction term between current ICU caseload and current ICU workload.

Several other studies have focused on potential effects intensive care staffing levels (as surrogates for workload) may have on outcomes of critically ill patients. While it is intuitive that higher patient-to-staff ratios lead to higher demand on individual health care professionals, findings regarding optimum ratios have varied between groups of healthcare professionals and have at times been conflicting.

Regarding physician staffing, a U-shaped correlation with an optimal patient-to-intensivist ratio of 7.5 has previously been reported in the United Kingdom [30]. However, no such association could be demonstrated in a study using data from Australia and New Zealand [31]. Regarding nursing staff levels, a linear increase in mortality with every additional patient cared for by a single ICU nurse has been reported in a study conducted in nine European countries [32]. Similarly, patient-to-nurse ratios above 1.5:1 have been found to be associated with increased mortality in an international cohort [33] and ratios above 2:1 have been found to be associated with increased resource use in patients treated in ICUs after surgery [34] or for cardiogenic shock [35].

We do not directly address staffing rates and ratios in this study. Information on on-scene staff from shift to shift is not available in the registry used. However, staffing requirements mirroring statements on the subject issued by professional entities [36–38] are mandated by Austrian national regulations. Given these fixed total staffing levels, we have focused on representatives of workload and caseload that could be influenced by overall capacity planning as well as admission and discharge policies, namely bed occupancy rates and workload intensity measured using TISS-28 [24].

Causes of worse outcomes due to excessive caseload and workload may be found both on unit levels and provider levels. For staff, strain may lead to the inability to provide care and perform interventions [39], suboptimal collaboration between staff groups due to stress and conflict [40], and ultimately burnout [41–43]. On unit and system levels, strain may lead to higher rates of discharge from ICU outside normal working hours [44], emergence of drug-resistant pathogens [45, 46], and ultimately “failure to rescue” [47].

Over-use of ICU capacities by means of utilising IMCU capacities directly connected to ICUs for the provision of critical care or even the creation of additional temporary capacities can occur both under normal working conditions and during surge or crises situations. We have observed over-use of ICU capacity, represented by current occupancy rates above one hundred per cent of planned bed capacities, not to be generally associated with a significantly higher probability of death in the ICU. This indicates that ICUs with mandated staffing levels are relatively robust to challenges presented to them during their operation. This finding is similar to a previous study [14] but contrasts with others [15–17]. A possible explanation could be lower acuity of patients in this Austrian patient cohort due to comparably higher ICU bed availabilities.

In contrast, over-use of ICU capacities has been associated with higher risk of death between 2020 and 2022, i.e., during the COVID-19 pandemic. This is in line with findings from other studies conducted during COVID-19 [6, 18, 19]. Moreover, current bed occupancy above one hundred per cent has been found to be associated with increased risk of death in ICUs situated at local hospitals, i.e., those with comparably low staff-to-bed ratios. These findings may be explained by the inability of teams and units to adapt and cope during situations dictated by circumstances rather than chosen by deliberation, especially when personnel reserves are comparably low, even in well-resourced health care systems.

We have also found high current workloads not to be generally associated with risk of death in the ICU. Intensive care units situated at local hospitals are again an exception, as median TISS-28 values of patients treated in these units of 30 and above have been found to be significantly associated with increased risk of death in ICU. These findings corroborate the notion that units designed to treat the most critically ill patients are well-equipped to deal with high workloads, whereas units meant to provide more basic intensive care may be overwhelmed by workload excesses.

We have also found under-use of ICU capacity, represented by current occupancy rates below fifty per cent of planned bed capacities, to be associated with a significantly higher risk of mortality. Explanations for this finding may be under-use of ICU capacities resulting in lower staff routine levels, the possibility to provide interventions that do not improve patient-oriented outcomes [48], or prolonged ICU admission of patients with low chances of survival that are more likely be treated elsewhere when demand is higher. This may be supported by the finding that low median workload over up to three days is not significantly associated with increased risk of death. Similarly, a previous study has reported ICU strain to be associated with shorter ICU stay [16].

### Strengths and limitations

This study was conducted in a large registry comprised of data contributed 144 ICUs mandated by national regulations, which warrants necessary completeness to address questions on a system-wide scale. Nevertheless, data stem from one nation only, potentially limiting the ability to apply findings to other nations and systems directly. Differences in intensive care provision, capacity, staffing, and utilisation may lead to different effects or breakpoints in elsewhere.

Both caseload and workload values reported in this study were possibly less extreme than in other systems, regions, and studies. The absence of significant effects of caseload and workload could be the result of participating ICUs working under "safe" circumstances according to their planning and staffing, similar to findings from earlier studies on workload [49].

Current workload was modelled using the well-validated TISS-28 [50–52]. TISS-28 may be inadequate to represent work of modern multi-professional ICU teams and current challenges, e.g., hyperactive delirium [53]. Median values within individual ICUs at a time allowed for good representation of workload irrespective of caseload. However, using median values as measures of central tendency may underrepresent peaks in workload, whereas mean values may overestimate them. We primarily chose median values as robust measures of central tendency.

Current caseload was represented by occupancy rates, which confers face validity. Modelling caseload in blocks of eight hours each allowed for representation of changes over the course of the day [26]. The use of maximum values within these time intervals may have led to overestimation of caseload in some instances. Available bed numbers were based on scheduled values used for remuneration and staff level calculations. Potential local deviations, such as simultaneous use of beds for ICU and IMCU purposes [54] as well as temporary bed closures [55], could not be accounted for. Inaccurate reporting of planned bed capacities could theoretically lead to bias.

## Conclusions

In a system with comparably high intensive care resources and mandatory staffing levels, patients’ chances of survival are generally not affected by high current intensive care unit caseload and workload. However, extraordinary circumstances, such as the COVID-19 pandemic, may lead to higher risk of death, if planned capacities are exceeded. High current workload in ICUs in smaller hospitals with lower staffing levels may be associated with increased risk of death. Clinicians and planners should thus be aware of units’ planned capacities as well as their current workload and caseload to adjust short-term and long-term planning accordingly.

## Supplementary Information

Additional file1.

## Data Availability

Anonymous data are available from the corresponding author upon reasonable request.

## Abbreviations

ASDI: Austrian Center for Documentation and Quality Assurance in Intensive Care Medicine
COVID: Coronavirus disease
ICU: Intensive care unit
IQR: Inter-quartile range
SAPS: Simplified Acute Physiology Score
TISS: Therapeutic Intervention Scoring System
