# Supplementary material for: Intensive care unit caseload and workload and their association with outcomes in critically unwell patients: a large registry-based cohort analysis
Source: Crit Care. 2024 Sep 14;28:304. doi: 10.1186/s13054-024-05090-z (PMC11401295; doi:10.1186/s13054-024-05090-z)
Supplement: Supplementary file 1 — Additional file1. [file 13054_2024_5090_MOESM1_ESM.pdf]

Supplementary Materials

Intensive Care Unit Caseload and Workload and their Association with Outcomes in Critically Unwell Patients: A Large Registry-Based Cohort Analysis

Paul ZAJIC, MD PhD[1]

Teresa ENGELBRECHT, BSc[2]

Alexandra GRAF, PhD[2]

Barbara METNITZ, PhD[3]

Rui MORENO, MD PhD[4]

Martin POSCH, PhD[2]

Andrew RHODES, MD[5]

Philipp METNITZ, MD PhD[1,3]

[1] Department of Anaesthesiology and Intensive Care Medicine, Medical University of Graz, Graz, Austria

[2] Center for Medical Data Science, Medical University of Vienna, Vienna, Austria

[3] Austrian Center for Documentation and Quality Assurance in Intensive Care, Vienna, Austria

[4] Hospital de São José, Unidade Local de Saúde São José, Lisbon, Portugal; Centro Clínico Académico de Lisboa, Lisbon, Portugal; Faculdade de Ciências da Saúde, Universidade da Beira Interior, Lisbon, Portugal

[5] Adult Critical Care, St. George's University Hospitals NHS Foundation Trust, St. George's University of London, London, United Kingdom

Sensitivity Analysis: Interaction Term between Workload and Caseload 2

Sensitivity Analysis: “Moving Median” Up to Three Days 4

Sensitivity Analysis: ICU Identifier as Covariate 5

Subgroup Analysis: Years 2013-2019 6

Subgroup Analysis: Years 2020-2022 7

Sensitivity Analysis: Interaction Terms for Years with Caseload and Workload 8

Subgroup Analysis: ICU Type “Surgical” 9

Subgroup Analysis: ICU Type “Medical” 10

Sensitivity Analysis: Interaction Terms for ICU Type with Caseload and Workload 11

Subgroup Analysis: Hospital Level “Primary Care Hospital” 12

Subgroup Analysis: Hospital Level “Secondary Care Hospital” 13

Subgroup Analysis: Hospital Level “Specialised Care Hospital” 14

Subgroup Analysis: Hospital Level “Tertiary Care Hospital” 15

Sensitivity Analysis: Interaction Terms for Hospital Levels with Caseload and Workload 16

Sensitivity Analysis: Interaction Term between Workload and Caseload

Table S 1 Cox proportional hazards regression with ICU mortality as endpoint. The main variable of interest, an interaction between workload based on the TISS-28-Score and caseload based on bed occupancy was modelled as a time-dependent covariable. SAPS3, sex, year of discharge, type of ICU, time of day, and calendar day were also included in the model and ICUID serves as a cluster variable

	HR	lower	upper	p-value
**SAPS3 [per point]**	1.05	1.05	1.06	< **0.0001**
**Hospital level: Reference: PRIM**				
Secondary care hospital	1.03	0.91	1.17	0.664
Specialised care hospital	0.74	0.62	0.87	< **0.0001**
Tertiary care hospital	0.91	0.74	1.12	0.366
**Time of the day: Reference: 8:00-15:59**				
16:00-23:59	0.90	0.86	0.94	< **0.0001**
00:00-7:59	0.49	0.47	0.52	< **0.0001**
**Type of ICU: Surgical vs Medical**	0.73	0.65	0.83	< **0.0001**
**Year of discharge: Reference: 2013**				
2014	0.92	0.86	0.99	**0.028**
2015	0.88	0.79	0.98	**0.016**
2016	0.98	0.86	1.11	0.699
2017	0.97	0.87	1.08	0.571
2018	1.00	0.89	1.13	0.943
2019	0.93	0.82	1.05	0.255
2020	1.12	0.99	1.26	0.080
2021	1.13	0.99	1.28	0.066
2022	1.03	0.92	1.15	0.590
**Current load based on current workload (TISS-28) & current caseload [%]: Reference: 21-30 & 76-100**				
≤ 20 ; 76-100	0.84	0.70	1.02	0.072
31 to 40 ; 76-100	0.99	0.91	1.08	0.861
> 40 ; 76-100	0.92	0.80	1.07	0.273
21 to 30 ; ≤ 50	1.06	0.96	1.17	0.255
≤ 20 ; ≤ 50	0.93	0.79	1.09	0.363
31 to 40 ; ≤ 50	1.10	0.99	1.21	0.078
> 40 ; ≤ 50	0.98	0.82	1.17	0.835
21 to 30 ; 51-75	1.02	0.95	1.09	0.581
≤ 20 ; 51-75	0.97	0.81	1.16	0.729
31 to 40 ; 51-75	1.12	1.01	1.24	**0.026**
> 40 ; 51-75	1.07	0.91	1.25	0.438
21 to 30 ; > 100	1.06	0.96	1.17	0.270
≤ 20 ; > 100	0.96	0.74	1.24	0.747
31 to 40 ; > 100	1.05	0.92	1.20	0.450
> 40 ; > 100	1.04	0.79	1.36	0.792
**Sex: Male vs. Female**	0.91	0.89	0.93	< **0.0001**
**Non-working day vs. working day**	0.89	0.86	0.91	< **0.0001**

Figure S 1 Results of a Cox proportional hazards regression with ICU mortality as endpoint. The main variable of interest, an interaction between workload based on the TISS-28-Score and caseload based on bed occupancy was modelled as a time-dependent covariable and is depicted here. SAPS3, sex, year of discharge, type of ICU, time of day, and calendar day were also included in the model and ICUID serves as a cluster variable

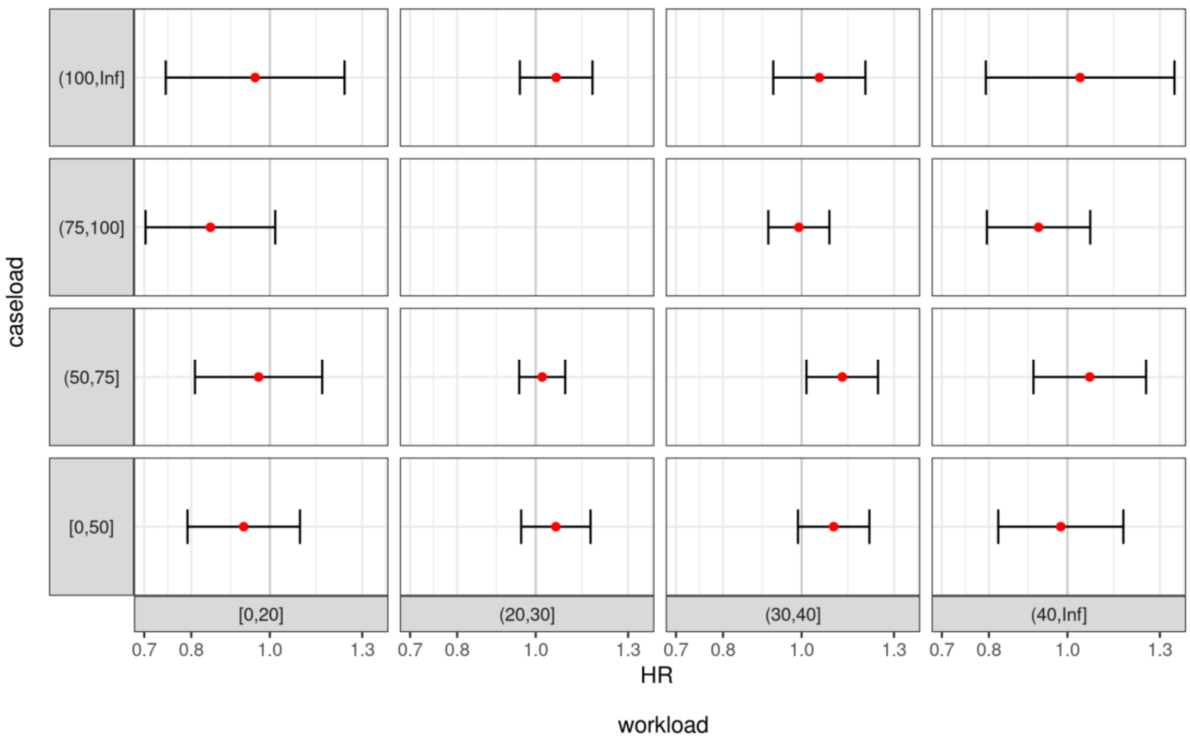

Sensitivity Analysis: “Moving Median” Up to Three Days

Table S 2 Cox proportional hazards regression with ICU mortality as endpoint. The main variables of interest, workload based on median TISS-28-Score over up to 3 days and caseload based on median bed occupancy up to 3 days were modelled as time-dependent covariables. SAPS3, sex, year of discharge, hospital level, type of ICU, time of day, and calendar day were also included in the model and ICUID serves as a cluster variable.

	HR	lower	upper	p-value
**SAPS3 [per point]**	1.05	1.05	1.06	**< 0.0001**
**Hospital level (Reference: Primary care hospital)**				
Secondary care hospital	1.02	0.90	1.15	0.794
Specialised care hospital	0.73	0.62	0.87	**< 0.0001**
Tertiary care hospital	0.89	0.73	1.09	0.259
**Time of day (Reference: 08:00-15:59)**				
16:00-23:59	0.91	0.87	0.94	**< 0.0001**
00:00-07:59	0.50	0.47	0.52	**< 0.0001**
**ICU type (Surgical vs. Medical)**	0.73	0.64	0.83	**< 0.0001**
**Year of discharge (Reference: 2013)**				
2014	0.92	0.85	0.99	**0.026**
2015	0.87	0.79	0.97	**0.013**
2016	0.97	0.86	1.10	0.675
2017	0.97	0.87	1.08	0.551
2018	1.00	0.89	1.13	0.936
2019	0.93	0.82	1.06	0.259
2020	1.12	0.99	1.27	0.065
2021	1.12	0.99	1.27	0.072
2022	1.04	0.93	1.16	0.469
**Moving median (3 days) of caseload (bed occupancy) [%]: Reference: 76-100**				
≤ 50	1.01	0.95	1.07	0.810
51-75	1.03	0.98	1.08	0.262
> 100	1.09	0.99	1.19	0.069
**Moving median (3 days) of workload of the others (TISS-28): (Reference: 21 to 30)**				
≤ 20	0.90	0.78	1.04	0.149
31 to 40	1.05	0.97	1.14	0.219
> 40	1.01	0.89	1.15	0.893
**Patient gender (Male vs. Female)**	0.91	0.89	0.94	**< 0.0001**
**Calendar day (Non-working day vs. working day)**	0.89	0.86	0.92	**< 0.0001**

Sensitivity Analysis: ICU Identifier as Covariate

Table S 3 Cox proportional hazards regression with ICU mortality as endpoint. The main variables of interest, workload based on median TISS-28-Score of up to 3 days and caseload based on median bed occupancy of up to 3 days were modelled as time-dependent covariables. SAPS3, sex, year of discharge, time of day, and calendar day were also included in the model. ICUID serves as a covariate, but is not depicted.

	HR	lower	upper	p-value
**SAPS3 score**	1.06			< **0.0001**
**Time of the day (Reference: 8:00-15:59)**				
16:00-23:59	0.90	0.88	0.92	< **0.0001**
00:00-7:59	0.49	0.48	0.51	< **0.0001**
**Year of discharge (Reference: 2013)**				
2014	0.92	0.88	0.97	**0.002**
2015	0.88	0.84	0.93	< **0.0001**
2016	0.91	0.87	0.96	< **0.0001**
2017	0.90	0.85	0.94	< **0.0001**
2018	0.92	0.88	0.97	**0.001**
2019	0.90	0.86	0.95	< **0.0001**
2020	1.03	0.98	1.08	0.186
2021	1.08	1.03	1.13	**0.002**
2022	1.00	0.95	1.06	0.883
**Current ICU Caseload (bed occupancy) [%]: Reference: 76-100**				
≤ 50	1.09	1.05	1.13	< **0.0001**
51-75	1.08	1.05	1.10	< **0.0001**
> 100	1.01	0.97	1.05	0.610
**Patient gender (Male vs. Female)**	0.90	0.88	0.92	< **0.0001**
**Current ICU workload (TISS-28): Reference: 21 to 30**				
≤ 20	0.92	0.86	0.99	**0.022**
31 to 40	1.00	0.98	1.03	0.739
> 40	0.95	0.89	1.01	0.085
**Calendar day (Non-working day vs. working day)**	0.88	0.86	0.90	< **0.0001**

Subgroup Analysis: Years 2013-2019

Table S 4 Cox proportional hazards regression with ICU mortality as endpoint. The main variables of interest, workload based on the TISS-28-Score and case-load based on bed occupancy were modelled as time-dependent covariables. SAPS3, sex, year of discharge, hospital level, type of ICU, time of day, and calendar day were also included in the model and ICUID serves as a cluster variable.

	HR	lower	upper	p-value
**SAPS3 [per point]**	1.05	1.05	1.06	**< 0.0001**
**Hospital level (Reference: Primary care hospital)**				
Secondary care hospital	1.06	0.93	1.21	0.412
Specialised care hospital	0.84	0.74	0.95	**0.006**
Tertiary care hospital	0.93	0.77	1.13	0.485
**Time of day (Reference: 08:00-15:59)**				
16:00-23:59	0.91	0.87	0.96	**< 0.0001**
00:00-07:59	0.51	0.48	0.54	**< 0.0001**
**ICU type (Surgical vs. Medical)**	0.74	0.65	0.85	**< 0.0001**
**Year of discharge (Reference: 2013)**				
2014	0.92	0.86	0.99	**0.031**
2015	0.88	0.79	0.98	**0.017**
2016	0.98	0.86	1.11	0.733
2017	0.97	0.87	1.08	0.599
2018	1.01	0.89	1.13	0.924
2019	0.93	0.82	1.05	0.240
**Current ICU caseload (bed occupancy) [%] (Reference: 76 to 100)**				
≤ 50	1.05	0.98	1.14	0.170
51-75	1.08	1.02	1.15	**0.006**
> 100	0.99	0.91	1.08	0.846
**Patient gender (Male vs. Female)**	0.91	0.88	0.94	**< 0.0001**
**Current ICU workload (TISS-28) [points] (Reference: 21 to 30)**				
≤ 20	0.87	0.76	1.00	**0.050**
31 to 40	1.02	0.94	1.11	0.632
> 40	0.93	0.81	1.06	0.279
**Calendar day (Non-working day vs. working day)**	0.89	0.86	0.92	**< 0.0001**

Subgroup Analysis: Years 2020-2022

Table S 5 Cox proportional hazards regression with ICU mortality as endpoint. The main variables of interest, workload based on the TISS-28-Score and case-load based on bed occupancy were modelled as time-dependent covariables. SAPS3, sex, year of discharge, hospital level, type of ICU, time of day, and calendar day were also included in the model and ICUID serves as a cluster variable.

	HR	lower	upper	p-value
**SAPS3 [per point]**	1.05	1.05	1.06	< **0.0001**
**Hospital level (Reference: Primary care hospital)**				
Secondary care hospital	0.97	0.83	1.13	0.690
Specialised care hospital	0.61	0.45	0.82	**0.001**
Tertiary care hospital	0.87	0.66	1.15	0.336
**Time of day (Reference: 08:00-15:59)**				
16:00-23:59	0.88	0.83	0.93	< **0.0001**
00:00-07:59	0.47	0.44	0.50	< **0.0001**
**ICU type (Surgical vs. Medical)**	0.72	0.62	0.84	< **0.0001**
**Year of discharge: Reference: 2020**				
2021	1.01	0.95	1.08	0.764
2022	0.93	0.85	1.03	0.157
**Current ICU caseload (bed occupancy) [%] (Reference: 76 to 100)**				
≤ 50	1.16	1.07	1.27	< **0.0001**
51-75	1.14	1.08	1.21	< **0.0001**
> 100	1.18	1.06	1.30	**0.001**
**Patient gender (Male vs. Female)**	0.91	0.88	0.95	< **0.0001**
**Current ICU workload (TISS-28) [points] (Reference: 21 to 30)**				
≤ 20	0.91	0.77	1.07	0.262
31 to 40	1.04	0.95	1.14	0.409
> 40	1.01	0.84	1.20	0.952
**Calendar day (Non-working day vs. working day)**	0.88	0.84	0.92	< **0.0001**

Sensitivity Analysis: Interaction Terms for Years with Caseload and Workload

Table S 6 Cox proportional hazards regression with ICU mortality as endpoint. The main variables of interest, workload based on the TISS-28-Score and case-load based on bed occupancy were modelled as time-dependent covariables. Interaction terms between years 2013-2019 and 2020-2022 with caseload and workload were also included. SAPS3, sex, year of discharge, type of ICU, time of day, and calendar day were also included in the model and ICUID serves as a cluster variable

	HR	lower	upper	p-value
**SAPS3 [per point]**	1.05	1.05	1.06	< **0.0001**
**Hospital level (Reference: Primary care hospital)**				
Secondary care hospital	1.02	0.90	1.16	0.776
Specialised care hospital	0.72	0.61	0.86	< **0.0001**
Tertiary care hospital	0.90	0.73	1.11	0.336
**Time of day (Reference: 08:00-15:59)**				
16:00-23:59	0.90	0.86	0.94	< **0.0001**
00:00-7:59	0.49	0.47	0.52	< **0.0001**
**ICU type (Surgical vs. Medical)**	0.73	0.64	0.83	< **0.0001**
**Years 2020 to 2022 vs. 2013 to 2019**	1.09	0.98	1.20	0.098
**Years 2013 to 2019 : Current ICU caseload (bed occupancy) [%] 76-100**				
2020-2022 ; ≤ 50	1.08	0.97	1.21	0.176
2020-2022 ; 51-75	1.04	0.96	1.13	0.305
2020-2022 ; > 100	1.19	1.06	1.34	**0.003**
**Current ICU caseload (bed occupancy) [%] (Reference: 76 to 100)**				
≤ 50	1.05	0.98	1.14	0.183
51-75	1.09	1.02	1.15	**0.006**
> 100	0.99	0.90	1.09	0.863
**Patient gender (Male vs. Female)**	0.91	0.89	0.94	< **0.0001**
**Current ICU workload (TISS-28) [points] (Reference: 21 to 30)**				
≤ 20	0.88	0.76	1.01	0.062
31 to 40	1.02	0.94	1.11	0.582
> 40	0.92	0.81	1.05	0.234
**Current ICU workload (TISS-28) [points] 21 to 30 : Years 2013 to 2019**				
≤ 20 ; 2020-2022	1.04	0.88	1.23	0.679
31 to 40 ; 2020-2022	1.01	0.93	1.11	0.757
>40 ; 2020-2022	1.09	0.91	1.32	0.335
**Calendar day (Non-working day vs. working day)**	0.89	0.86	0.92	< **0.0001**

Subgroup Analysis: ICU Type “Surgical”

Table S 7 Cox proportional hazards regression with ICU mortality as endpoint. The main variables of interest, workload based on the TISS-28-Score and case-load based on bed occupancy were modelled as time-dependent covariables. SAPS3, sex, year of discharge, hospital level, time of day, and calendar day were also included in the model and ICUID serves as a cluster variable.

	HR	lower	upper	p-value
**SAPS3 [per point]**	1.06	1.06	1.06	**< 0.0001**
**Hospital level (Reference: Primary care hospital)**				
Secondary care hospital	1.05	0.91	1.21	0.479
Specialised care hospital	0.72	0.57	0.91	**0.005**
Tertiary care hospital	0.96	0.78	1.17	0.656
**Time of day (Reference: 08:00-15:59)**				
16:00-23:59	0.89	0.84	0.94	**< 0.0001**
00:00-07:59	0.46	0.43	0.50	**< 0.0001**
**Year of discharge (Reference: 2013)**				
2014	0.91	0.84	0.99	**0.033**
2015	0.92	0.84	1.01	0.066
2016	1.01	0.90	1.13	0.924
2017	0.95	0.84	1.07	0.402
2018	0.98	0.84	1.14	0.766
2019	0.90	0.76	1.07	0.231
2020	1.12	0.96	1.31	0.139
2021	1.12	0.95	1.33	0.190
2022	1.04	0.91	1.20	0.544
**Current ICU caseload (bed occupancy) [%] (Reference: 76 to 100)**				
≤ 50	1.10	1.02	1.19	**0.012**
51-75	1.09	1.04	1.13	**< 0.0001**
> 100	1.02	0.92	1.13	0.658
**Patient gender (Male vs. Female)**	0.90	0.88	0.93	**< 0.0001**
**Current ICU workload (TISS-28) [points] (Reference: 21 to 30)**				
≤ 20	0.99	0.86	1.13	0.828
31 to 40	0.99	0.93	1.06	0.837
> 40	0.96	0.83	1.11	0.571
**Calendar day (Non-working day vs. working day)**	0.89	0.85	0.94	**< 0.0001**

Subgroup Analysis: ICU Type “Medical”

Table S 8 Cox proportional hazards regression with ICU mortality as endpoint. The main variables of interest, workload based on the TISS-28-Score and case-load based on bed occupancy were modelled as time-dependent covariables. SAPS3, sex, year of discharge, hospital level, time of day, and calendar day were also included in the model and ICUID serves as a cluster variable.

	HR	lower	upper	p-value
**SAPS3 [per point]**	1.05	1.04	1.05	**< 0.0001**
**Hospital level (Reference: Primary care hospital)**				
Secondary care hospital	1.03	0.83	1.27	0.818
Specialised care hospital	0.78	0.65	0.93	**0.006**
Tertiary care hospital	0.90	0.61	1.32	0.596
**Time of day (Reference: 08:00-15:59)**				
16:00-23:59	0.91	0.86	0.97	**0.006**
00:00-07:59	0.53	0.50	0.57	**< 0.0001**
**Year of discharge (Reference: 2013)**				
2014	0.93	0.83	1.04	0.210
2015	0.84	0.70	1.00	0.055
2016	0.94	0.76	1.16	0.569
2017	0.99	0.84	1.16	0.867
2018	1.02	0.86	1.22	0.800
2019	0.96	0.80	1.15	0.648
2020	1.10	0.92	1.31	0.320
2021	1.11	0.92	1.33	0.270
2022	1.01	0.85	1.20	0.886
**Current ICU caseload (bed occupancy) [%] (Reference: 76 to 100)**				
≤ 50	1.05	0.96	1.16	0.292
51-75	1.11	1.02	1.20	**0.011**
> 100	1.10	0.99	1.23	0.090
**Patient gender (Male vs. Female)**	0.92	0.89	0.96	**< 0.0001**
**Current ICU workload (TISS-28) [points] (Reference: 21 to 30)**				
≤ 20	0.80	0.68	0.95	**0.010**
31 to 40	1.09	0.96	1.23	0.190
> 40	0.94	0.79	1.12	0.497
**Calendar day (Non-working day vs. working day)**	0.87	0.84	0.91	**< 0.0001**

Sensitivity Analysis: Interaction Terms for ICU Type with Caseload and Workload

Table S 9 Cox proportional hazards regression with ICU mortality as endpoint. The main variables of interest, workload based on the TISS-28-Score and caseload based on bed occupancy were modelled as time-dependent covariables. Interaction terms between ICU Types with caseload and workload were also included. SAPS3, sex, year of discharge, type of ICU, time of day, and calendar day were also included in the model and ICUID serves as a cluster variable

	HR	lower	upper	p-value
**SAPS3 [per point]**	1.05	1.05	1.06	**< 0.0001**
**Hospital level (Reference: Primary care hospital)**				
Secondary care hospital	1.03	0.91	1.17	0.672
Specialised care hospital	0.73	0.62	0.86	**< 0.0001**
Tertiary care hospital	0.91	0.74	1.12	0.356
**Time of day (Reference: 08:00-15:59)**				
16:00-23:59	0.90	0.86	0.94	**< 0.0001**
00:00-7:59	0.49	0.47	0.52	**< 0.0001**
**ICU type (Surgical vs. Medical)**	0.74	0.63	0.88	**< 0.0001**
**ICU Type Medical : Current ICU caseload (bed occupancy) [%] 76-100**				
Surgical ; ≤ 50	1.05	0.92	1.20	0.472
Surgical ; 51-75	0.98	0.90	1.08	0.705
Surgical ; > 100	0.88	0.75	1.04	0.140
**Year of discharge (Reference: 2013)**				
2014	0.92	0.86	0.99	**0.031**
2015	0.88	0.79	0.98	**0.016**
2016	0.98	0.86	1.11	0.724
2017	0.97	0.87	1.08	0.579
2018	1.00	0.89	1.13	0.946
2019	0.93	0.82	1.06	0.260
2020	1.12	0.99	1.26	0.071
2021	1.13	1.00	1.28	0.060
2022	1.03	0.92	1.16	0.563
**Current ICU caseload (bed occupancy) [%] (Reference: 76 to 100)**				
≤ 50	1.05	0.95	1.17	0.311
51-75	1.11	1.02	1.20	**0.011**
> 100	1.15	1.01	1.30	**0.031**
**Patient gender (Male vs. Female)**	0.91	0.89	0.93	**< 0.0001**
**Current ICU workload (TISS-28) [points] (Reference: 21 to 30)**				
≤ 20	0.85	0.72	1.00	**0.048**
31 to 40	1.04	0.92	1.17	0.541
> 40	0.89	0.74	1.08	0.239
**Current ICU workload (TISS-28) (20,30] : ICU Type Medical**				
≤ 20 ; Surgical	1.12	0.91	1.39	0.273
31 to 40 ; Surgical	0.99	0.86	1.13	0.837
> 40 ; Surgical	1.11	0.88	1.41	0.366
**Calendar day (Non-working day vs. working day)**	0.89	0.86	0.91	**< 0.0001**

Subgroup Analysis: Hospital Level "Primary Care Hospital"

Table S 10 Cox proportional hazards regression with ICU mortality as endpoint. The main variables of interest, workload based on the TISS-28-Score and caseload based on bed occupancy were modelled as time-dependent covariables. SAPS3, sex, year of discharge, type of ICU, time of day, and calendar day were also included in the model and ICUID serves as a cluster variable.

	HR	lower	upper	p-value
**SAPS3 [per point]**	1.06	1.06	1.06	**< 0.0001**
**Time of day (Reference: 08:00-15:59)**				
16:00-23:59	0.92	0.87	0.97	**0.002**
00:00-07:59	0.60	0.53	0.69	**< 0.0001**
**ICU type (Surgical vs. Medical)**	0.65	0.53	0.79	**< 0.0001**
**Year of discharge (Reference: 2013)**				
2014	0.92	0.75	1.14	0.455
2015	1.02	0.80	1.32	0.847
2016	1.06	0.84	1.34	0.638
2017	1.11	0.89	1.39	0.354
2018	1.13	0.92	1.39	0.240
2019	0.99	0.80	1.21	0.903
2020	1.26	1.00	1.58	0.052
2021	1.35	1.08	1.70	**0.009**
2022	1.31	1.05	1.64	**0.019**
**Current ICU caseload (bed occupancy) [%] (Reference: 76 to 100)**				
≤ 50	1.06	0.99	1.14	0.074
51-75	1.03	0.97	1.10	0.334
> 100	1.08	0.97	1.20	0.160
**Patient gender (Male vs. Female)**	0.98	0.92	1.04	0.494
**Current ICU workload (TISS-28) [points] (Reference: 21 to 30)**				
≤ 20	0.93	0.81	1.06	0.250
31 to 40	1.09	1.01	1.19	**0.035**
> 40	1.28	1.02	1.60	**0.033**
**Calendar day (Non-working day vs. working day)**	0.96	0.88	1.04	0.273

Subgroup Analysis: Hospital Level “Secondary Care Hospital”

Table S 11 Cox proportional hazards regression with ICU mortality as endpoint. The main variables of interest, workload based on the TISS-28-Score and caseload based on bed occupancy were modelled as time-dependent covariables. SAPS3, sex, year of discharge, type of ICU, time of day, and calendar day were also included in the model and ICUID serves as a cluster variable.

	HR	lower	upper	p-value
**SAPS3 [per point]**	1.05	1.05	1.06	**< 0.0001**
**Time of day (Reference: 08:00-15:59)**				
16:00-23:59	0.91	0.86	0.97	**0.004**
00:00-07:59	0.48	0.46	0.51	**< 0.0001**
**ICU type (Surgical vs. Medical)**	0.75	0.65	0.88	**< 0.0001**
**Year of discharge (Reference: 2013)**				
2014	0.96	0.88	1.04	0.324
2015	0.87	0.76	0.99	**0.042**
2016	0.93	0.79	1.10	0.394
2017	0.92	0.81	1.05	0.203
2018	0.96	0.83	1.12	0.622
2019	0.92	0.79	1.08	0.313
2020	1.05	0.91	1.20	0.512
2021	1.12	0.96	1.32	0.149
2022	1.03	0.90	1.18	0.683
**Current ICU caseload (bed occupancy) [%] (Reference: 76 to 100)**				
≤ 50	1.09	1.00	1.19	0.056
51-75	1.13	1.06	1.20	**< 0.0001**
> 100	1.05	0.96	1.15	0.300
**Patient gender (Male vs. Female)**	0.90	0.87	0.92	**< 0.0001**
**Current ICU workload (TISS-28) [points] (Reference: 21 to 30)**				
≤ 20	0.75	0.54	1.03	0.073
31 to 40	0.98	0.89	1.08	0.685
> 40	0.90	0.78	1.04	0.143
**Calendar day (Non-working day vs. working day)**	0.88	0.84	0.91	**< 0.0001**

Subgroup Analysis: Hospital Level “Specialised Care Hospital”

Table S 12 Cox proportional hazards regression with ICU mortality as endpoint. The main variables of interest, workload based on the TISS-28-Score and caseload based on bed occupancy were modelled as time-dependent covariables. SAPS3, sex, year of discharge, type of ICU, time of day, and calendar day were also included in the model and ICUID serves as a cluster variable.

	HR	lower	upper	p-value
**SAPS3 [per point]**	1.06	1.05	1.06	**< 0.0001**
**Time of day (Reference: 08:00-15:59)**				
16:00-23:59	1.01	0.91	1.11	0.919
00:00-07:59	0.35	0.25	0.48	**< 0.0001**
**ICU type (Surgical vs. Medical)**	0.71	0.56	0.90	**0.005**
**Year of discharge (Reference: 2013)**				
2014	1.09	0.95	1.25	0.239
2015	0.83	0.66	1.05	0.120
2016	1.03	0.90	1.18	0.703
2017	0.99	0.85	1.16	0.913
2018	0.98	0.83	1.16	0.842
2019	0.92	0.60	1.39	0.680
2020	0.89	0.41	1.93	0.763
2021	0.80	0.55	1.17	0.245
2022	0.99	0.75	1.31	0.948
**Current ICU caseload (bed occupancy) [%] (Reference: 76 to 100)**				
≤ 50	1.01	0.82	1.26	0.911
51-75	1.00	0.85	1.19	0.964
> 100	1.03	0.77	1.38	0.855
**Patient gender (Male vs. Female)**	0.84	0.78	0.90	**< 0.0001**
**Current ICU workload (TISS-28) [points] (Reference: 21 to 30)**				
≤ 20	0.95	0.75	1.19	0.626
31 to 40	0.84	0.68	1.04	0.105
> 40	0.78	0.56	1.08	0.131
**Calendar day (Non-working day vs. working day)**	0.82	0.70	0.97	**0.017**

Subgroup Analysis: Hospital Level “Tertiary Care Hospital”

Table S 13 Cox proportional hazards regression with ICU mortality as endpoint. The main variables of interest, workload based on the TISS-28-Score and caseload based on bed occupancy were modelled as time-dependent covariables. SAPS3, sex, year of discharge, type of ICU, time of day, and calendar day were also included in the model and ICUID serves as a cluster variable.

	HR	lower	upper	p-value
**SAPS3 [per point]**	1.05	1.04	1.06	**< 0.0001**
**Time of day (Reference: 08:00-15:59)**				
16:00-23:59	0.78	0.70	0.86	**< 0.0001**
00:00-07:59	0.45	0.39	0.52	**< 0.0001**
**ICU type (Surgical vs. Medical)**	0.69	0.42	1.13	0.140
**Year of discharge (Reference: 2013)**				
2014	0.77	0.63	0.93	**0.006**
2015	0.84	0.64	1.08	0.176
2016	1.18	0.82	1.71	0.366
2017	1.11	0.77	1.62	0.572
2018	1.16	0.77	1.76	0.471
2019	0.98	0.58	1.64	0.932
2020	1.45	0.97	2.18	0.072
2021	1.24	0.80	1.91	0.336
2022	0.75	0.55	1.03	0.073
**Current ICU caseload (bed occupancy) [%] (Reference: 76 to 100)**				
≤ 50	1.11	0.91	1.35	0.310
51-75	1.12	1.03	1.21	**0.006**
> 100	1.05	0.85	1.29	0.678
**Patient gender (Male vs. Female)**	0.88	0.83	0.94	**< 0.0001**
**Current ICU workload (TISS-28) [points] (Reference: 21 to 30)**				
≤ 20	1.12	0.79	1.59	0.519
31 to 40	1.16	0.87	1.54	0.307
> 40	1.04	0.73	1.48	0.817
**Calendar day (Non-working day vs. working day)**	0.84	0.78	0.90	**< 0.0001**

Sensitivity Analysis: Interaction Terms for Hospital Levels with Caseload and Workload

Table S 14 Cox proportional hazards regression with ICU mortality as endpoint. The main variables of interest, workload based on the TISS-28-Score and caseload based on bed occupancy were modelled as time-dependent covariables. Interaction terms between Hospital Levels with caseload and workload were also included. SAPS3, sex, year of discharge, type of ICU, time of day, and calendar day were also included in the model and ICUID serves as a cluster variable

	HR	lower	upper	p-value
**SAPS3 [per point]**	1.05	1.05	1.06	< **0.0001**
**Time of day (Reference: 08:00-15:59)**				
Secondary care hospital	1.11	0.93	1.32	0.231
Specialised care hospital	0.92	0.75	1.13	0.425
Tertiary care hospital	0.88	0.71	1.09	0.251
**Hospital level: Primary care hospital & Current ICU Caseload (bed occupancy) [%]: 76-100**				
Secondary care hospital ; ≤ 50	0.99	0.88	1.11	0.808
Specialised care hospital ; ≤ 50	0.89	0.66	1.21	0.460
Tertiary care hospital ; ≤ 50	1.02	0.83	1.25	0.835
Secondary care hospital ; 51-75	1.08	0.99	1.17	0.101
Specialised care hospital ; 51-75	0.96	0.78	1.17	0.660
Tertiary care hospital ; 51-75	1.05	0.95	1.16	0.339
Secondary care hospital ; > 100	0.99	0.86	1.14	0.907
Specialised care hospital ; > 100	0.89	0.63	1.24	0.481
Tertiary care hospital ; > 100	0.96	0.73	1.26	0.753
**Time of day (Reference: 08:00-15:59)**				
16:00-23:59	0.90	0.86	0.94	< **0.0001**
00:00-7:59	0.49	0.47	0.52	< **0.0001**
**ICU type (Surgical vs. Medical)**	0.73	0.65	0.83	< **0.0001**
**Year of discharge: Reference: 2013**				
2014	0.92	0.86	0.99	**0.027**
2015	0.88	0.79	0.98	**0.017**
2016	0.98	0.86	1.11	0.727
2017	0.97	0.88	1.08	0.621
2018	1.01	0.90	1.13	0.890
2019	0.93	0.82	1.06	0.269
2020	1.12	1.00	1.27	0.058
2021	1.14	1.00	1.29	**0.046**
2022	1.04	0.93	1.16	0.515
**Current ICU caseload (bed occupancy) [%] (Reference: 76 to 100)**				
≤ 50	1.09	1.01	1.18	**0.020**
51-75	1.04	0.98	1.11	0.194
> 100	1.07	0.97	1.19	0.185
**Patient gender (Male vs. Female)**	0.91	0.89	0.93	< **0.0001**
**Current ICU workload (TISS-28) [points] (Reference: 21 to 30)**				
≤ 20	0.93	0.82	1.06	0.260
31 to 40	1.13	1.04	1.22	**0.006**
> 40	1.31	1.05	1.63	**0.018**

	HR	lower	upper	p-value
**Hospital level: Primary Care Hospital & Current ICU Workload (TISS-28): 21 to 30**				
Secondary care hospital ; ≤ 20	0.82	0.58	1.14	0.232
Secondary care hospital ; 31 to 40	0.86	0.76	0.99	**0.031**
Secondary care hospital ; >40	0.68	0.52	0.89	**0.005**
Specialised care hospital ; ≤ 20	1.00	0.76	1.32	0.976
Specialised care hospital ; 31 to 40	0.75	0.61	0.92	**0.006**
Specialised care hospital ; > 40	0.62	0.44	0.86	**0.005**
Tertiary care hospital ; ≤ 20	1.26	0.91	1.77	0.168
Tertiary care hospital ; 31 to 40	1.01	0.83	1.23	0.917
Tertiary care hospital ; > 40	0.74	0.54	1.03	0.071
**Calendar day (Non-working day vs. working day)**	0.89	0.86	0.91	**< 0.0001**
